# Ethno-Medicinal Plants Used to Cure Jaundice by Traditional Healers of Mashhad, Iran

**Published:** 2014

**Authors:** Mohammad Sadegh Amiri, Mohammad Reza Joharchi, Mohammad Ehsan TaghavizadehYazdi

**Affiliations:** a *Department of Biology, Payame Noor University, Tehran, Iran. *; b *Ferdowsi University of Mashhad, Mashhad. Iran. *; c *Department of Plant Sciences, Eram Biotechnology Research Center, Technical and Vocational Training Organization, Mashad,. Iran.*

**Keywords:** Ethnomedicinal, Jaundice, Traditional healers, Mashhad, Iran

## Abstract

Jaundice is the commonest ailments affecting the citizens of both developed and poor Asians countries including Iran. An ethnobotanical survey of plants used by the traditional healers for the treatment of jaundice was conducted in the Mashhad city, Northeastern Iran. A total of 37 plants belonging to 32 genera and 26 families have been documented for their therapeutic use against jaundice. The plant families which contained the most commonly used species for their effects are: Fabaceae (5 species), Polygonaceae (4 sp.), Asteraceae (3 sp.), Plantaginaceae (2 sp.) and Salicaceae (2 sp.). The plants were arranged with correct nomenclature along with their common name, family, the part used and their medicinal value. The use of decoction is the most preferred method of herbal preparation. In all cases, the treatment involved oral administration of the extracts 2 to 3 times daily from a week to month till the problem disappears. *Cichorium intybus*, *Salix alba*, *Cotoneaster nummularius*, *Descurainia sophia*, *Malva sylvestris*, *Berberis integrrima*, *Rumex acetosella*, *Phyllanthus emblica* and *Alhagi maurorum *were repeatedly mentioned by the traditional healers as the most widely used for the treatment of jaundice in the study area. The study indicates that the local inhabitants rely on medicinal plants for treatment. This paper suggested that further clinical experimentation is needed to scientifically evaluate these widely used herbal remedies for possible bioactive effects.

## Introduction

During the last few decades there has been an increasing interest in the study of medicinal plants and their traditional use in different parts of the world (1). Documenting the indigenous knowledge through ethnobotanical studies is not only useful for conservation of cultural traditions and biodiversity but also for community healthcare and drug development in the present and future (2). Ethnobotanical data supply clues for materials to be tested by clinical and pharmacological researches, provide new distribution areas for raw drugs and a broad base for interaction with other systems of medicines. However, of the estimated 350,000 plant species worldwide only a small percentage has been investigated phytochemically and an even smaller percentage has been properly studied in terms of their pharmacological properties (3). Today according to the World Health Organization (WHO), as many as 80% of the world's people depend on traditional medicine for their primary healthcare needs. There are considerable economic benefits in the development of indigenous medicines and in the use of medicinal plants for the treatment of various diseases (4). Among different ailments, jaundice is the commonest ailments affecting the citizens of the world countries including Iran. Jaundice is the yellowish staining of the skin and sclera (the whites of the eyes) that is caused by high levels in blood of the chemical bilirubin. The color of the skin and sclera vary depending on the level of bilirubin. When the bilirubin level is mildly elevated, they are yellowish. When the bilirubin level is high, they tend to be brown. Jaundice may result from various diseases or conditions that affect the liver, like Hepatitis A, Hepatitis B, Hepatitis C, Hepatitis D, Hepatitis E, Autoimmune hepatitis, Liver cirrhosis, liver cancer, Hemolytic anaemia and Malaria (5). Our precedential study among the traditional healers (named Attar) of Mashhad city indicated that jaundice was one of the major problems among them and they used herbal remedies to heal it. Although some ethnobotanical studies have been accomplished among Iranian people by some earlier researchers, including Hooper (6), Ghorbani (7), Amiri (8) and Rajaei (9); no-systematic ethnotherapeutic studies have been conducted to evaluate of the traditional remedies of jaundice. The present investigation was undertaken with the aim of producing an inventory of the plants used by traditional healers to document ethnomedical information on potentially valuable medicinal plants for the development of new pharmaceuticals and also to emphasize the role of ethnomedicine to cure icterus.

## Experimental

*Study area and ethnic people*

The study was conducted during 2011–12 under the project “Assessment of traditional medicinal plants commercialized in the markets of Mashhad, Iran”, to elicit data on medicinal plants used by traditional healers of this metropolis for the treatment of jaundice. Mashhad is situated at the northeast of Iran. It is the second largest city in Iran and one of the holiest cities in the world. Its approximate geographic location is 35°43' to 37°8' north latitude and 59°15' to 60°36' east longitude, in the valley of the Kashaf River near Turkmenistan, between the two mountain ranges of Binalood and Hezar-masjed. It is located in the center of the Razavi Khorasan province close to the borders of Afghanistan and Turkmenistan. The total area of the Mashhad is 270 km^2^ and the population of the city is about 3 million people. There are also over 20 million pilgrims who visit the city every year. The vast majority of the Mashhad people are ethnic Persians who form over 95% of the city's population. Other ethnic groups include Kurdish and Turkmen people who have immigrated recently to the city from the North Khorasan province.

*Data collection*

During the course of exploration of ethnomedicinal plants the information including the various data such as name and age of informants, local names, purpose of usage, preparation procedure and duration of the treatment were obtained from traditional healers through discussions and questionnaires were used to gather their knowledge. A totally more than 100 informants with in the age group of 37 to 82 were interviewed, these included males and females. Subsequently, scientific identification and authentication was made with the help of Flora Iranica (10), Flora of Iran (11) and consulting with different herbal literature (12, 13). After correct identification, the specimens were deposited at the Ferdowsi University of Mashhad Herbarium (FUMH) for future references. For data analysis, plant species were tabulated and grouped into their respective families along with the relevant information. In this paper, all of data were updated on the base of latest changes of plant molecular systematic according to the rules of the International Code of Botanical Nomenclature (www.theplantlist.org website).

## Results and Discussion

**Figure 1:**
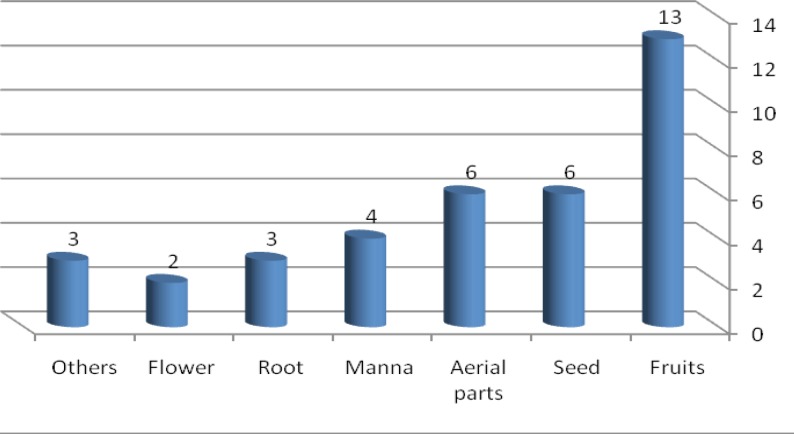
Distribution of medicinal plants parts used in the treatment of jaundice

The present study demonstrated that traditional healers used 37 species of ethnomedicinal plants (distributed in 32 genera belonging to 26 families) to cure jaundice. From the point of view of Taxonomy, plants that are being used for therapeutic purpose in this region belong to divisions of Pteridophyta (one species from Polypodiaceae and Pteridaceae) and Magnoliophyta (with two classes Magnoliopsida and Liliopsida, including 24 families all together). According to Table 1, Maximum number of medicinal plant species belongs to family Fabaceae (5 species) followed by Polygonaceae (4 sp.), Asteraceae (3 sp.), Plantaginaceae (2 sp.) and Salicaceae (2 sp.). The rest of the families are represented by one species only. Data obtained from this survey is compiled in Table 1 and the folklore medicinal plants are arranged in alphabetic order. For each plant species botanical name, family, vernacular name, parts used, preparation and application are provided. Different plant parts were used to cure jaundice. Among these fruits were reported to be the most used part of the plants, constituting 35% of the herbal preparations. This was followed by the seed (16%), aerial parts (16%), manna (11%), root (8%) and flower (6%) (Figure 1). 

**Figure 2:**
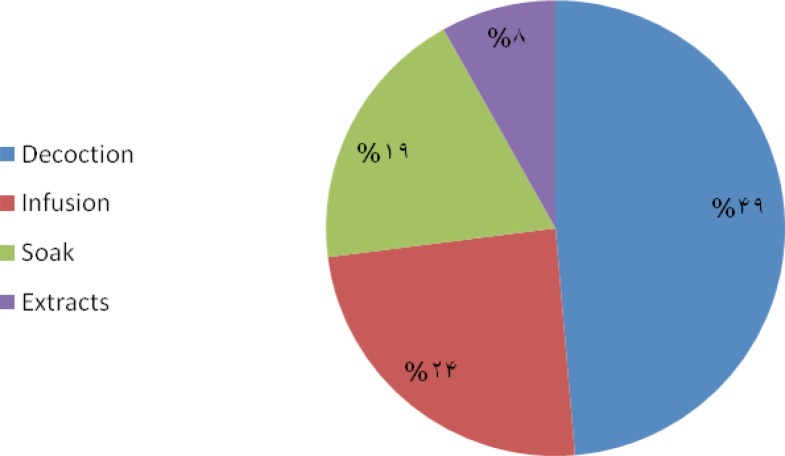
Preparation method of medicinal plants and their percentages

The methods of preparation are divided into four categories, among which the commonest method was decoction. Plant parts are used in the form of decoction (47%), infusion (26%), soak (19%) and extracts (8%) from the various parts of the plant (Figure 2). 

In all the cases mode of application was oral. In regard to the patient conditions, the preparation is usually administered two to three times per day from a week to month till the problem disappears. Icterus among Asians, especially Iranians is common. Some of the plant species include *Adiantum capillus-veneris*,* Rheum* *ribes*,* Salix* *alba*, *Cotoneaster nummularius*,* Tribulus* *terrestris, Descurainia sophia*, *Malva Phyllanthus emblica*, *Plantago ovata*, *Rhus coriaria*,* Tamarandus indica*,* Fumaria* *vaillantii* and* Alhagi* *maurorum* were repeatedly mentioned by the traditional healers as the most widely used for the treatment of adults jaundice in the study area. Jaundice is one of the most common problems found in a neonate which appears during the first week of life. Hyperbilirubinemia may develop serious complications like kernicterus and lifelong disability (14). There are two usual methods of treatment for neonatal jaundice in the literatures: light therapy and blood exchange (15). Blood exchange is the last way for decreasing the serum bilirubin levels (16). Furthermore, practical management by giving traditional remedies like *Alhagi graecorum*, *Cotoneaster nummularius* and *Descurainia sophia* extracts to breast-fed babies for reducing jaundice is popular in Iranian culture (17). This problem was obviously easily diagnosed by the old herbalists. General physical condition and tongue or eyes color of the patient are used as indicators of the patient’s problem. Six plants were frequently recommended by the traditional healers of the study area which demonstrated excellent results for the treatment of newborn jaundice. The most efficient are: *Rumex* *acetosella*, *Cichorium* *intybus*, *Alhagi graecorum*, *Cotoneaster nummularius, Cassia* *fistula* *and Alhagi* *maurorum*. This study determined that phytotherapy is the most common form of treatment for jaundice which showed significant results based on their use of traditional medicine in this area.

**Table 1:** Medicinal plants used for the treatment of jaundice by local inhabitants

**NO**	**Family Name**	**Botanical Name**	**Vernacular** **Name**	**Part used**	**Preparation**	**Ethnomedicinal Uses**	**Remarks**
1	Anacardiaceae	*Rhus* *coriaria* L*.*	Somagh	Fruit	Infusion	**Jaundice**, Cholesterol Lowering, Diabetes, Antihypertensive, Anti-hemorrhage, Flavoring	Indigenous
2	Apiaceae	*Coriandrum* *sativum* L.	Geshniz	Fruit	Infusion	**Jaundice**, Acne, Appetizer, Treat of Flatulence, Calmative, Antiseptic, Aromatic	Indigenous
3	Asteraceae	*Cichorium* *intybus* L*.*	Kasni	Aerial parts	Decoction	**Jaundice**, Treat of Palpitation, Appetizer, Depurative, Treat of Febrifuge, Antiallergic	Indigenous
4	Asteraceae	*Cynara* *scolymus* L.	Kangar Farangi	Aerial parts	Decoction	**Jaundice**, Liver Tonic, Digestive	Imported
5	Asteraceae	Silybum marianum (L.) Gaertn.	Khare Maryam	Seed	Decoction	**Jaundice**, Liver Tonic, Antihepatit, Febrifuge	Indigenous
6	Beberidaceae	*Berberis integrrima *Bunge	Zereshk Kuhi	Fruit	Extracts	**Jaundice**, Hypoglycemic, Antihypertensive, Blood and Liver Cleanser, Febrifuge	Indigenous
7	Brassicaceae	Descurainia sophia (L.) Webb ex Prantl	Khakshir	Seed	Soak	**Jaundice**, Blood and Liver Cleanser, Febrifuge, Laxative, Treat of Furuncles, Anti-thirst	Indigenous
8	Capparaceae	*Capparis* *spinosa* L*.*	Kavar	Fruit	Decoction	**Jaundice**, Liver Tonic, Hepatitis, Appetizer, Anthelmintic, Emmenagogue, Antigout	Indigenous
9	Combretaceae	Terminalia chebula Retz.	Halileh Siah	Fruit	Decoction	**Jaundice**, Purgative, Treat of Constipation, Liver Tonic	Imported
10	Cyperaceae	*Cyperus* *rotundus* L.	Soade Kufi	Root	Decoction	**Jaundice**, Strengthening of Memory	Indigenous
11	Fabaceae	*Alhagi graecorum* Boiss.	Taranjabin	Manna	Soak	**Jaundice**, Laxative, Febrifuge, Thirst, Aphthous Ulcers	Indigenous
12	Fabaceae	* Alhagi maurorum *Medik*.*	Khar Shotor- Taranjabin	Aerial parts - Manna	Decoction- Soak	**Jaundice**, Appetite Suppressant, Diuretic, Febrifuge	Indigenous
13	Fabaceae	Astragalus *fasciculifolius* subsp. *arbusculinus* (Bornm. & Gauba)Tietz	Anzerut	Gum	Decoction	**Jaundice**, Antitussive, Laxative, Anthelmintic	Indigenous
14	Fabaceae	*Cassia* *fistula* L.	Folus	Fruit	Extracts	**Jaundice**, Treat of Leishmaniasis, Infant Colic, Febrifuge, Purgative	Imported
15	Fabaceae	*Tamarindus* *indica* L*.*	Tamr Hendi	Fruit	Decoction	**Jaundice**, Depurative, Pimples	Imported
16	Lamiaceae	Salvia macrosiphon Boiss.	Kenocheh	Seed	Infusion	**Jaundice**, Gastric ulcer, Pharyngitis, Antitussive	Indigenous
17	Malvaceae	*Malva* *sylvestris* L*.*	Panirak	Flower	Infusion	**Jaundice**, Pharyngitis, Furuncles, Aphthous Ulcers, Antitussive	Indigenous
18	Papaveraceae	*Fumaria* *vaillantii* Loisel.	Shatareh	Aerial parts	Infusion	**Jaundice**, Psoriasis, Appetizer, Antiacid, Febrifuge	Indigenous
19	Phyllanthaceae	*Phyllanthus* *emblica* L.	Agheleh Moghashar	Fruit	Decoction	**Jaundice**, Diabetes, Febrifuge, Blood flux	Imported
20	Plantaginaceae	*Plantago* *major* L*.*	Barhang	Seed	Soak	**Jaundice**, Eczema, Antiallergic, Febrifuge, Antitussive	Indigenous
21	Plantaginaceae	Plantago ovata Forssk.	Esfarzeh	Seed	Soak	**Jaundice**, Obesity, Depilator, Tonsillitis, Antacid, Antitussive	Indigenous
22	Polygonaceae	*Rheum* *palmatum* L*.*	Rivand Chini	Root	Decoction	**Jaundice**, Liver Diseases, Cardiac Tonic, Antilithiasis	Imported
23	Polygonaceae	*Rheum* *ribes *L.	Rivas	Fruit	Infusion	**Jaundice**, Urinary Antiseptic, Diuretic, Depurative, Liver Tonic	Indigenous
24	Polygonaceae	Rheum turkestanicum Janisch.	Eshghan	Root	Extracts	**Jaundice**, Diabetes, Antihypertensive, Depurative	Indigenous
25	Polygonaceae	*Rumex* *acetosella* L*.*	Sagh Torshak	Aerial parts	Decoction	**Jaundice**, Febrifuge	Indigenous
26	Polypodiaceae	*Polypodium* *vulgare* L*.*	Baspayak	Rhizome	Infusion	**Jaundice**, Expectorant	Indigenous
27	Portulacaceae	*Portulaca* *oleracea* L.	Khorfeh	Seed	Soak	**Jaundice**, Antitussive, Febrifuge, Anti-Thirst, Depurative	Indigenous
28	Primulaceae	Embelia ribes Burm.f.	Barang Kaboli	Fruit	Decoction	**Jaundice**, Anthelmintic, Antidiarrhea	Imported
29	Pteridaceae	Adiantum capillus-veneris L.	Parsiavashan	Frond	Infusion	**Jaundice**, Antitussive, Antihemorrhoid, Febrifuge	Indigenous
30	Rhamnaceae	*Ziziphus* *jujuba *Miller	Annab	Fruit	Decoction	**Jaundice**, Depurative, Febrifuge	Indigenous
31	Rosaceae	Cotoneaster nummularius Fisch. & C.A.Mey.	Shir Khesht	Manna	Soak	**Jaundice**, Febrifuge	Indigenous
32	Rutaceae	*Toddalia* *asiatica* (L.) Lam*.*	Dahanbaz-Dahan basteh	Fruit	Decoction	**Jaundice**, Diabetes, Febrifuge	Imported
33	Salicaceae	*Salix* *alba* L*.*	Bid	Leaves	Decoction	**Jaundice**, Menstrual Pains, Anodyne, Antitussive	Indigenous
34	Salicaceae	Salix excelsa J.F.Gmel.	Bidkhesht	Manna	Soak	**Jaundice**, Febrifuge, Laxative	Indigenous
35	Solanaceae	*Physalis* *alkekengi* L.	Arusak Posht Pardeh	Fruit	Decoction	**Jaundice**, Emmenagogue, Treat of Kidney Stones	Indigenous
36	Violaceae	Viola odorata L.	Banafsheh	Flower	Infusion	**Jaundice**, Eczema, Febrifuge, Antiallergic, Blood Cleansing	Indigenous
37	Zygophyllaceae	*Tribulus* *terrestris* L*.*	Kharkhasak	Aerial parts	Decoction	**Jaundice**, Duretic, Kidney Stone, Tonic, Treatment of Prostate Hypertrophy, Anthelmintic	Indigenous

## Conclusion

The present investigation revealed that the study area have a variety of medicinal plants which are used by the local inhabitants for their primary healthcare. The results depicts that traditional healers used 37 species of ethnomedicinal plants to cure jaundice. Among them, 29 species were indigenous of Iran and 8 species were imported from other countries. By comparing these plant species recorded for the treatment of icterus with available pharmacological literature reported from other regions of the world, it appears that there are many medicinal plant species in the area that were not reported in other locations. To our knowledge the use of *Rheum turkestanicum*, *Physalis* *alkekengi, Toddalia* *asiatica*, *Salvia macrosiphon *and *Salix excels* to cure jaundice, have never been reported before. These ethnomedicinal data may provide a baseline to start the search the new compounds related to pharmacology, pharmacognosy and phytochemistry. Therefore, the survey ascertains the value of a great number of medicinal plants used in traditional remedies against icterus which could be of considerable interest in the development of new drugs in the future. 
